# Metagenomic Next-Generation Sequencing of Cerebrospinal Fluid for the Diagnosis of External Ventricular and Lumbar Drainage-Associated Ventriculitis and Meningitis

**DOI:** 10.3389/fmicb.2020.596175

**Published:** 2020-12-14

**Authors:** Lingye Qian, Yijun Shi, Fangqiang Li, Yufei Wang, Miao Ma, Yanfang Zhang, Yang W. Shao, Guanghui Zheng, Guojun Zhang

**Affiliations:** ^1^Department of Clinical Diagnosis, Laboratory of Beijing Tiantan Hospital, Capital Medical University, Beijing, China; ^2^Nanjing Geneseeq Technology Inc., Nanjing, China; ^3^School of Public Health, Nanjing Medical University, Nanjing, China

**Keywords:** metagenomic next-generation sequencing (mNGS), diagnosis, EVD, LD, ventriculitis and meningitis

## Abstract

Metagenomic next-generation sequencing (mNGS) has become a widely used technology that can accurately detect individual pathogens. This prospective study was performed between February 2019 and September 2019 in one of the largest clinical neurosurgery centers in China. The study aimed to evaluate the performance of mNGS on cerebrospinal fluid (CSF) from neurosurgical patients for the diagnosis of external ventricular and lumbar drainage (EVD/LD)-associated ventriculitis and meningitis (VM). We collected CSF specimens from neurosurgical patients with EVD/LD for more than 24 h to perform conventional microbiological studies and mNGS analyses in a pairwise manner. We also investigated the usefulness of mNGS of CSF for the diagnosis of EVD/LD-associated VM. In total, 102 patients were enrolled in this study and divided into three groups, including confirmed VM (cVM) (39), suspected VM (sVM) (49), and non-VM (nVM) (14) groups. Of all the patients, mNGS detected 21 Gram-positive bacteria, 20 Gram-negative bacteria, and five fungi. The three primary bacteria detected were *Staphylococcus epidermidis* (9), *Acinetobacter baumannii* (5), and *Staphylococcus aureus* (3). The mNGS-positive coincidence rate of confirmed EVD/LD-associated VM was 61.54% (24/39), and the negative coincidence rate of the nVM group was 100% (14/14). Of 15 VM pathogens not identified by mNGS in the cVM group, eight were negative with mNGS and seven were inconsistent with the conventional microbiological identification results. In addition, mNGS identified pathogens in 22 cases that were negative using conventional methods; of them, 10 patients received a favorable clinical treatment; thus, showing the benefit of mNGS-guided therapy.

## Introduction

External ventricular drains (EVDs) and, in some specific cases, lumbar drains (LDs) are essential to drain cerebrospinal fluid (CSF) and control intracranial pressure after neurosurgery (Brain Trauma Foundation et al., 2007; Carney et al., 2017). One of the risks surrounding the placement and maintenance of catheters is the infection of cerebrospinal fluid (CSF), with infection rates ranging from 2 to 45% (Lozier et al., 2008; Scheithauer et al., 2010; van de Beek et al., 2010; Dorresteijn et al., 2019). EVD/LD-associated ventriculitis and meningitis (VM) are correlated with high rates of morbidity and mortality, significantly prolonged lengths of hospitalization (LOS), elevated hospital costs, and negatively affected prognosis (Citerio et al., 2015; Hussein et al., 2019). However, the diagnosis and pathogen identification of VM samples can be difficult (Ross et al., 1988), causing delays in treatment, and the administration of inadequate/inappropriate antimicrobial therapies (Phan et al., 2016).

Metagenomic next-generation sequencing (mNGS) is a high-throughput sequencing technology that could overcome the limitations of conventional diagnostic tests and allow for hypothesis-free, culture-independent pathogen detection directly from biological specimens (Simner et al., 2018). mNGS provides a comprehensive method that accurately identifies all potential pathogens, including viruses, bacteria, fungi, and parasites in a single test (Simner et al., 2018). This approach is valuable in distinguishing pathogens that cause infections with non-specific and overlapping clinical manifestations (Miller et al., 2019). Recent advances in mNGS have facilitated the development of rapid and user-friendly data analysis tools, and the creation of accurate and comprehensive databases. Combined with its low cost, mNGS has become a first-line laboratory method in response to emerging infectious diseases and outbreaks. mNGS has also become increasingly popular in routine clinical practice for the rapid identification of pathogens in CNS infections from CSF (Guan et al., 2016; Fan et al., 2018; Wilson et al., 2019; Xing et al., 2019). However, most published studies use non-standardized methods and lack standards for verifications. In addition, inadequate CSF samples and the lack of relevant studies on EVD/LD patients also hinder the broad application of mNGS in the diagnosis of VM.

What remains to be explored is whether the diagnosis of EVD/LD-associated VM using mNGS is as effective and efficient as conventional microbiological methodologies for pathogen detection and identification. Here, we compare diagnostic methods in 102 neurosurgical patients with EVD/LD and assess the performance of mNGS in identifying CSF pathogens and predicting VM. We also evaluate whether mNGS can provide therapeutic guidance for EVD/LD-associated VM, using *Acinetobacter baumannii* and *Stenotrophomonas maltophilia*-induced VM as examples.

## Materials and Methods

This study was performed at the Beijing Tiantan Hospital and Capital Medical University between February 2019 and September 2019. Beijing Tiantan Hospital is a tertiary hospital with 1,850 beds and more than 15,000 annual surgeries. The protocol for this study was approved by the ethical committee of Beijing Tiantan Hospital and Capital Medical University (Approval Number: KY-2019-095-03).

### Diagnostic Criteria and Data Abstraction

Neurosurgical patients 18 years of age and older with an EVD/LD for more than 24 h were eligible. Patients with EVDs or LDs inserted for cerebral or spinal infective processes were excluded. Patients who were discharged without a new surgery died within 7 days after neurosurgery, or those with an inadequate CSF volume (<0.5 ml) or incomplete medical records were also excluded from the study. All patients enrolled in the study were followed up with throughout the first 30 postoperative days.

Since there are no universally accepted definitions of device-related VM, for the purpose of this study, we defined the diagnosis criteria as follows (Leib et al., 1999; Horan et al., 2008; Citerio et al., 2015): (1) confirmed VM (cVM) was characterized by a positive CSF culture and a CSF leukocyte count >250 cells/μl; (2) suspected VM (sVM) was characterized by a CSF leukocyte count >1,000 cells/μl (neutrophil percentage >50%) or a CSF neutrophil count >250 cells/μl; and (3) non-VM (nVM) was defined by a negative CSF culture and CSF leukocyte count <250 cells/μl, with <50% neutrophils.

All patients' daily progress notes were extracted from the standard database. Nineteen clinical features were included, such as patient characteristics (age, gender), hypertension, diabetes mellitus, fever (>38°C), LOS, time of infection clearance, Glasgow Coma Scale (GCS), CSF leakage, craniotomy, surgical wound classification, reoperation, assist mechanical ventilation, tumor, intensive care unit (ICU) admission, bacteremia, hospital-acquired pneumonia, mortality, and clinical treatment, including antibiotic prophylaxis, empirical use of antibiotics, and definitive therapy. In addition, we recorded 12 clinical laboratory indicators, including CSF cell count (C-Cell), CSF leukocyte count (C-Leu), CSF neutrophil percentage (C-Neu), CSF protein concentration (C-Pro), CSF chloride ion concentration (C-Cl), CSF glucose concentration (C-Glu), CSF lactate concentration (C-Lac), blood leukocyte count (B-Leu), blood glucose concentration (B-Glu), blood neutrophil percentage (B-Neu), the ratio of C-Glu to B-Glu (C/B-Glu), and procalcitonin level (PCT). All of the clinical laboratory parameters were obtained on the same day that EVD/LD-associated VM occurred.

### mNGS of CSF

#### Sample Processing

CSF specimens were subjected to conventional microbiological studies (e.g., culture, serology) and pairwise mNGS. Specimens (0.5 to 1.2 ml) were stored at −80°C until use. Fresh CSF was extracted for routine clinical examination. Antibiotic resistance was determined from bacterial genome sequences using mNGS.

#### Nucleic Acid Extraction

CSF samples were collected from patients according to standard procedures. DNA was extracted using the TIANamp Magnetic DNA kit (Tiangen) according to the manufacturer's protocols. The quantity and quality of DNA were assessed using Qubit (Thermo Fisher Scientific) and NanoDrop (Thermo Fisher Scientific) instruments, respectively.

#### Library Preparation and Sequencing

DNA libraries were prepared using the KAPA Hyper Prep kit (KAPA Biosystems) according to the manufacturer's protocols. An Agilent 2100 was used for quality control, and single-read sequencing of 75 bp DNA libraries was sequenced on an Illumina Next-Seq 550Dx (Illumina).

#### Bioinformatics Analysis

An in-house-developed bioinformatics pipeline was used for pathogen identification. Briefly, high-quality sequencing data were obtained following the removal of low-quality reads, adapter contamination, and duplicated and short (read length <36 bp) reads. Human host sequences were identified by mapping to the human reference genome (hs37d5) using bowtie2 software. Reads that could not be mapped to the human genome were retained and aligned with the NCBI microorganism genome database for pathogen identification. The microorganism genome database is composed of more than 20,000 NCBI reference genomes of bacteria, fungi, viruses, and parasites (download from ftp://ftp.ncbi.nlm.nih.gov/genomes/genbank/). The mapped reads were taxonomically classified using the Expectation Maximization algorithm.

#### Interpretation and Reporting

**Figure 1:**
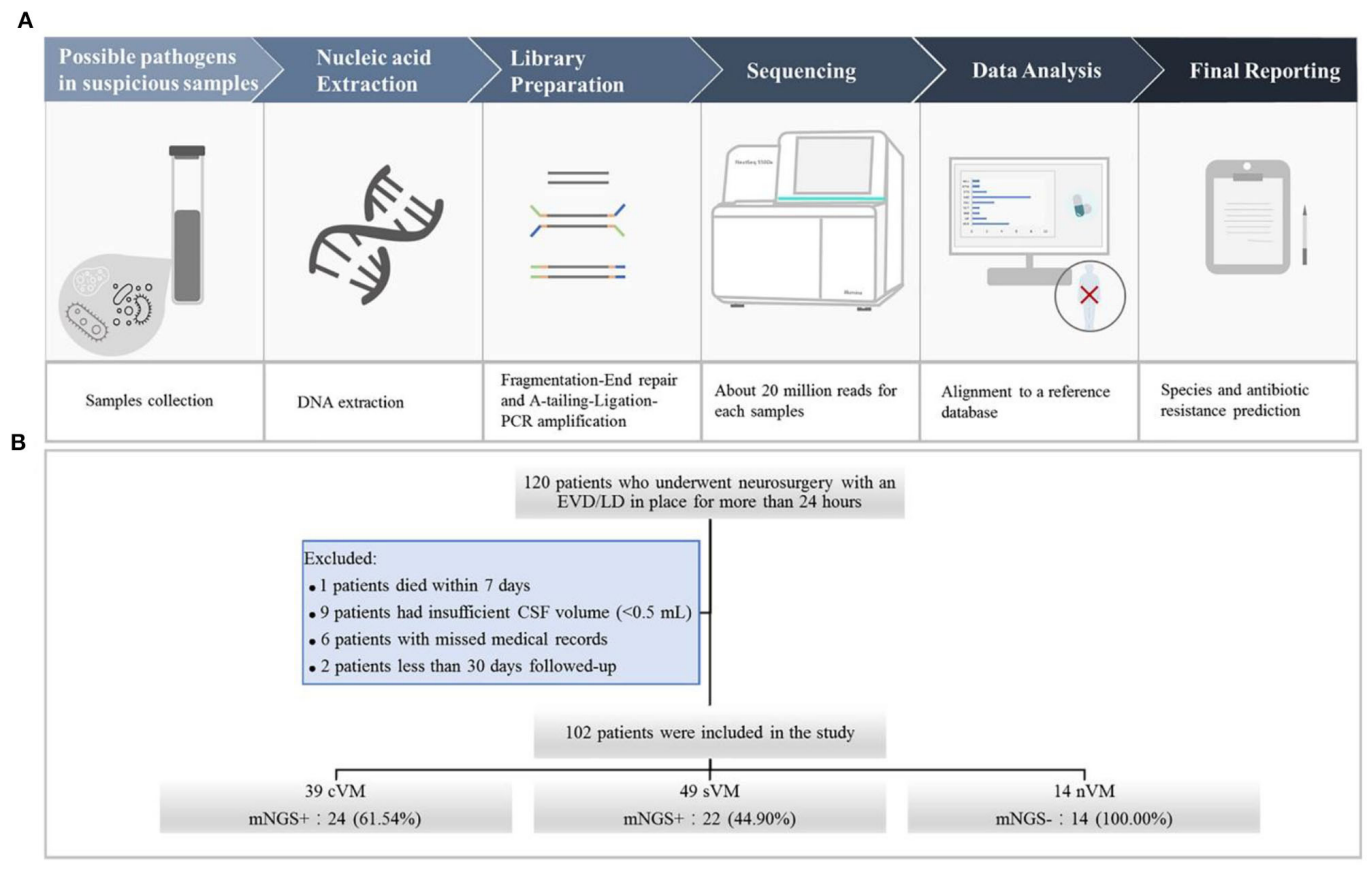
**(A)** Schematic of the mNGS workflow. After collecting CSF samples, DNA was extracted using the TIANamp Magnetic DNA Kit (Tiangen). DNA libraries were prepared using the KAPA Hyper Prep kit (KAPA Biosystems) and fragmentation-end repair and a-tail-ligation PCR amplification. Libraries were quantified, pooled, and loaded onto the sequencer. Using an in-house-developed bioinformatics pipeline, high-quality sequencing data were obtained. Reads were aligned to the NCBI microorganism genome database for pathogen identification. Finally, we reported the results of mNGS according to the criteria. **(B)** Flowchart of study participants.

We used the following criteria for the positive results from mNGS: (1) For *Mycobacterium, Nocardia*, and *Legionella pneumophila*, the results were considered positive if more than one unique read mapped to the same species. (2) For bacteria (excluding *Mycobacterium, Nocardia*, and *Legionella pneumophila*), fungi, virus, and parasites, the result was considered positive for a specific species if at least three unique, non-overlapping reads mapped to that species. (3) Pathogens that were detected in the negative “no-template” control (NTC) were excluded if the number of detected reads was <10-fold of that in the NTC. The schematic of the mNGS workflow is shown in Figure 1A.

#### Analysis of Antibiotic Resistance Genes

Megahit v1.2.5 was used to assemble reads and remove the host sequences using default parameters. Identification of the resulting contigs was performed by matching them to CARD v3.0.5 using RGI v5.1.0. Potential resistance genes were filtered using the following parameters: identity of 84%, and query coverage of 90%.

### Statistical Analysis

SAS 15.0 and EXCEL 2010 were used for statistical analyses. The distribution of continuous variables was described using the mean and standard deviation (SD), or median and interquartile range (IQR). Categorical variables were described as counts and percentages. For continuous quantitative variables, the Mann–Whitney *U*-test was performed to compare two groups and the Kruskal–Wallis H test was used to compare three or more groups. The Fisher's exact test was performed to compare categorical data. The original contributions presented in the study are publicly available. This data can be found at https://www.ncbi.nlm.nih.gov/sra/PRJNA660207.

## Results

### Clinical and Laboratory Data Representation

A total of 120 patients who underwent neurosurgery and had an EVD/LD in place for more than 24 h were screened. Of those, one patient died within 7 days, nine patients had insufficient CSF volumes (<0.5 mL), six patients had incomplete medical records, and two patients were followed up for <30 days. A total number of 102 CSF specimens were obtained from the remaining 102 patients, including 39 cVM, 49 sVM, and 14 nVM patients (Figure 1B).

The median age of the 102 patients was 41 years, and 59 patients were male (57.84%). The median LOS was 20.5 days (range, 15–27), and the median time to infection clearance was 6 days (range, 3–10.5). Eighteen patients (17.65%) had hypertension, two (1.96%) had diabetes mellitus, 23 (22.55%) experienced fever, and 11 (10.78%) reported CSF leakage. The incidence of craniotomy surgery and reoperation was 68.32% (69/102) and 14.71% (15/102), respectively, and 62.75% of cases (66/102) had clean surgical wounds after the procedure, while the remainder had clean-contaminated surgical wounds. Of all patients, 80 (78.43%) had tumors and 29 (27.88%) were admitted to the ICU. Other common comorbidities included hospital-acquired pneumonia (8/102, 7.84%) and bacteremia (5/102, 4.90%). The majority of patients received antibiotics. During the 30-day follow-up period, two patients died of VM. The median C-cell count was 3,841.5 cells/μL (range, 1,432–8,897), median C-Leu count was 1,577.5 cells/μL (range, 289–3,651), and median C-Neu count was 83.45% (range, 74.5–89.3). All demographic and clinical characteristics are summarized in Table 1. Univariate analysis was conducted to evaluate the association of the above clinical features with the VM groups. Of those, 10 characteristics showed significant differences among the three groups, including age (*P* = 0.03), LOS (*P* = 0.04), craniotomy (*P* < 0.01), reoperation (*P* = 0.03), assisted mechanical ventilation (*P* < 0.01), bacteremia (*P* = 0.02), received definitive therapy (*P* < 0.01), C-Cell count (*P* < 0.01), C-Leu count (*P* < 0.01), and C-Neu count (*P* = 0.01).

**Table 1:** Demographic and clinical characteristics of the 102 patients.

**Characteristics**	**All patients (102)**	**cVM (39)**	**sVM (49)**	**Non-VM (14)**	***P*-value**	**P1**	**P2**
Age (median, IQR, years)	41 (29, 55)	45 (39, 57)	41 (29, 55)	30 (15, 40)	0.03	<0.01	0.07
Gender (male, %)	59 (57.84%)	22 (56.41%)	26 (53.06%)	11 (78.57%)	0.25	0.20	0.13
Hypertension	18 (17.65%)	10 (25.64%)	7 (14.28%)	1 (7.14%)	0.26	0.25	0.67
Diabetes mellitus	2 (1.96%)	1 (2.46%)	1 (2.04%)	0 (0%)	1.00	1.00	1.00
Fever (b.t >38°C)	23 (22.55%)	13 (33.33%)	8 (16.32%)	2 (14.28%)	0.14	0.30	1.00
LOS (median, IQR)	20.5 (15, 27)	24(16, 31)	18 (15, 24)	22.5 (18, 27)	0.04	0.75	0.12
Time of cure of infection (median, IQR)	6 (3, 10.5)	6 (3, 11)	4 (1, 5)	6.5 (3.5, 12.0)	0.37	0.88	0.21
GCS (median, IQR)	5 (4, 9)	5 (4.0, 10.5)	–	7.1 (7.1, 7.1)	0.70	0.70	–
CSF leakage	11 (10.78%)	4 (10.25%)	7 (14.28%)	0 (0%)	0.45	0.56	0.33
Craniotomy	70 (68.63%)	18 (46.15%)	39 (79.59%)	13 (92.85%)	<0.01	<0.01	0.43
Surgical wound classification					0.78	1.00	1.00
Clean (I)	64 (62.75%)	26 (66.67%)	29 (59.18%)	9 (64.29%)			
Clean-contaminate (II)	38 (37.25%)	13 (33.33%)	20 (40.82%)	5 (35.71%)			
Reoperation	15 (14.71%)	10 (25.64%)	3 (6.12%)	2 (14.29%)	0.03	0.48	0.31
Assist mechanical ventilation	15 (14.71%)	13 (33.33%)	0 (0%)	2 (14.29%)	<0.01	0.30	0.05
Tumor	80 (78.43%)	31 (79.49%)	37 (75.51%)	12 (85.71%)	0.85	1.00	0.72
ICU admission	29 (27.88%)	14 (35.89%)	11 (22.45%)	4 (28.57%)	0.37	0.75	0.73
Bacteremia	5 (4.90%)	5 (12.82%)	0 (0%)	0 (0%)	0.02	0.31	\
Hospital-acquired pneumonia	8 (7.84%)	6 (15.38%)	1 (2.04%)	1 (7.14%)	0.05	0.66	0.40
Mortality	2 (1.96%)	2 (5.13%)	0 (0%)	0 (0%)	0.40	1.00	\
**Antibiotics**							
Antibiotic prophylaxis	84 (82.35%)	36 (92.31%)	36 (73.47%)	12 (85.71%)	1.00	0.60	0.49
Empirical therapy	91 (89.22%)	35 (89.73%)	43 (87.76%)	13 (92.86%)	1.00	1.00	1.00
Definitive therapy	82 (80.39%)	37 (94.87%)	34 (69.38%)	11 (78.57%)	<0.01	0.11	0.74
C-cell/μl (median, IQR)	3,841.5 (1,432, 8,897)	3,186 (671, 8,109)	4,671 (2,548, 14,442)	806.5 (125, 3,695)	<0.01	0.07	<0.01
C-leu/μl (median, IQR)	1,577.5 (289, 3,651)	586 (242, 2,110)	2,310 (1,632, 4,396)	171.5 (51,535)	<0.01	<0.01	<0.01
C-neu% (median, IQR)	83.45 (74.5, 89.3)	84 (66.9, 89.3)	85.7 (79.6, 89.9)	65.5 (27.7, 83.6)	0.01	0.07	<0.01
C-pro mg/dl (median, IQR)	121.305 (84.58, 212.03)	112.2 (84.6, 230.8)	139.6 (94.9, 194.9)	81.4 (65.8, 191.5)	0.12	0.17	0.04
C-Cl mmol/L (median, IQR)	119.5 (115, 123)	121.0 (115.0, 125.0)	118.7 (114.7, 120.6)	120.6 (117.3, 123.8)	0.16	0.81	0.20
C-GLU mmol/L (median, IQR)	2.4 (1.65, 3.15)	2.6 (1.9, 3.7)	2.1 (1.4, 3.1)	2.6 (1.2, 3.1)	0.14	0.44	0.83
C-lac mmol/L (median, IQR)	5.35 (3.8, 6.3)	5.4 (3.4, 7.0)	5.5 (4.8, 6.3)	4.6 (2.4, 5.3)	0.10	0.14	0.03
B-leu mg/L (median, IQR)	11.84 (9.02, 14.97)	11.7 (8.8, 15.3)	11.9 (9.2, 15.0)	12.1 (8.2, 14.1)	0.92	0.83	0.95
B-glu mmol/L (median, IQR)	6.13 (5.22, 7.38)	6.1 (5.0, 8.0)	6.3 (5.3, 7.4)	5.9 (5.3, 7.0)	0.79	0.98	0.62
B-neu% (median, IQR)	8.85 (7.15, 12.19)	8.9 (7.0, 12.2)	8.7 (7.5, 12.4)	9.4 (5.7, 11.8)	0.93	0.98	0.91
C/B-glu (median, IQR)	0.36 (0.2, 0.49)	0.4 (0.3, 0.6)	0.4 (0.2, 0.4)	0.4 (0.2, 0.5)	0.30	0.32	0.88
PCT (median, IQR)	0.11 (0.0, 0.59)	0.1 (0.0, 0.7)	0.1 (0.0, 0.1)	–	0.92	–	–

*P1, Comparison between cVM and nVM; P2, Comparison between sVM and nVM*.

### Use of mNGS to Diagnose EVD/LD-Associated VM

**Figure 2:**
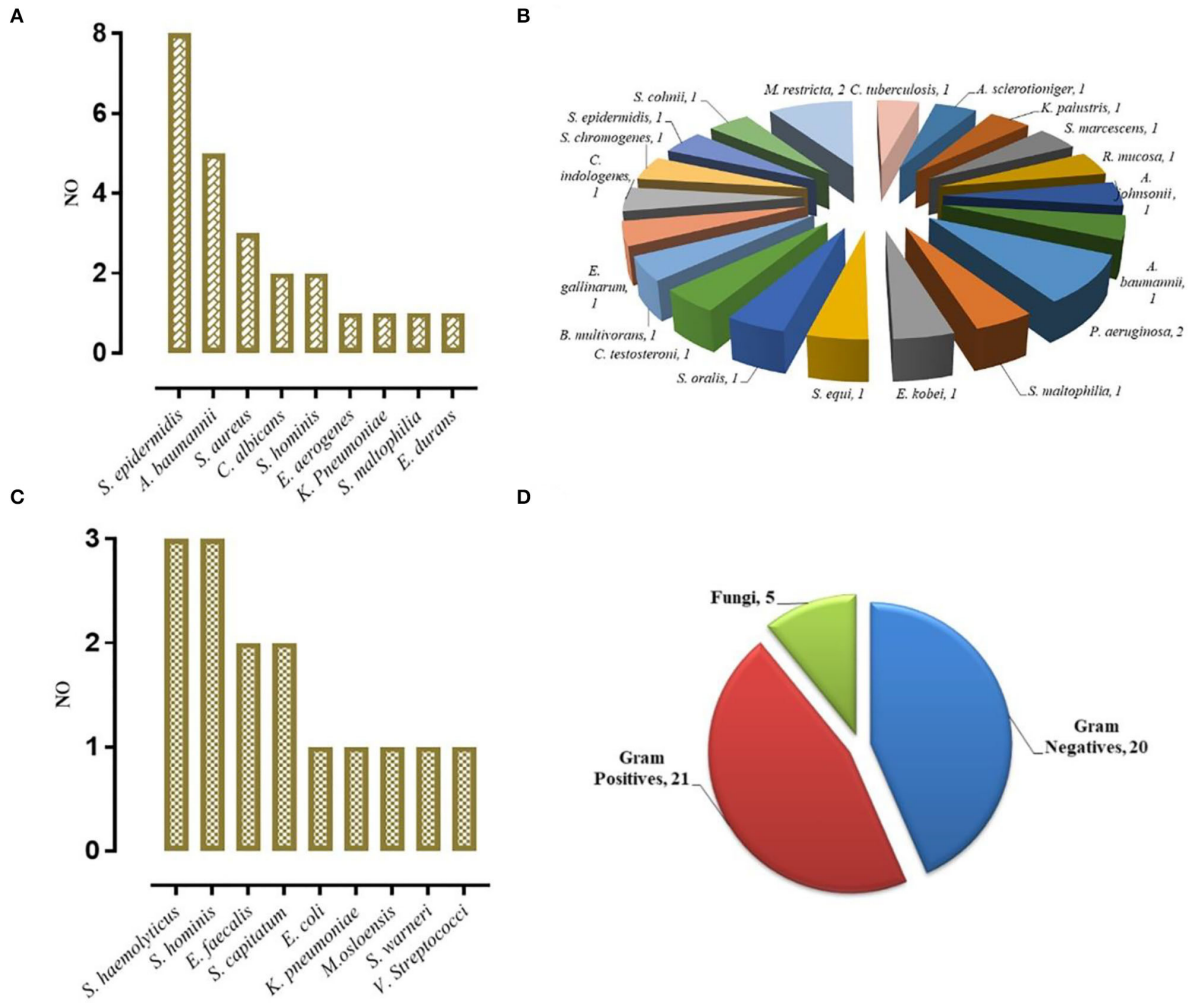
VM Diagnosed by mNGS. **(A)** Concurrent diagnosis by mNGS and conventional microbiological testing (24). **(B)** Diagnosis by mNGS only (22). **(C)** Diagnosis by conventional testing only (15). **(D)** The category of pathogens diagnosed by mNGS.

To study the effectiveness and efficiency of mNGS for pathogen identification in VM patients, 102 CSF samples were tested by mNGS and conventional microbiological identification. Among the 88 patients diagnosed with cVM (*n* = 39) and sVM (*n* = 49), 24 (27.27%) were diagnosed by conventional identification and mNGS (Figure 2A), 22 (25.00%) were diagnosed by mNGS only (Figure 2B), 15 (17.05%) were diagnosed by conventional testing only (Figure 2C), and the remaining 27 (30.68%) were not diagnosed by either method. Fourteen nVM patients showed negativity using both methods. Notably, all mNGS results were returned in <48 h, if not within 24 h. Such a turnaround was significantly shorter than that obtained using conventional microbiological tests, especially for positive samples.

Different types of infectious agents were identified by mNGS. Of the patients diagnosed by mNGS, 45.65% of VMs were caused by Gram-positive bacteria, 43.48% were caused by Gram-negative bacteria, and 10.87% were caused by fungi (Figure 2D). Furthermore, mNGS detected three fungal species, including *Candida albicans, Aspergillus sclerotioniger*, and *Malassezia restricta*. Among the 21 Gram-positive bacteria identified, *Staphylococcus epidermidis* (8, 38.10%) was the most common, followed by *Staphylococcus aureus* (3, 14.29%). Conversely, *A. baumannii* (5, 25.00%) was the most common Gram-negative bacterium identified. In addition, some pathogens were detected solely by mNGS, such as *Pseudomonas aeruginosa, Serratia marcescens, Acinetobacter johnsonii*, and *Burkholderia multivorans*, which highlighted the advantage of mNGS over conventional methods as such pathogens are not typically considered by clinicians during routine practice.

**Figure 3:**
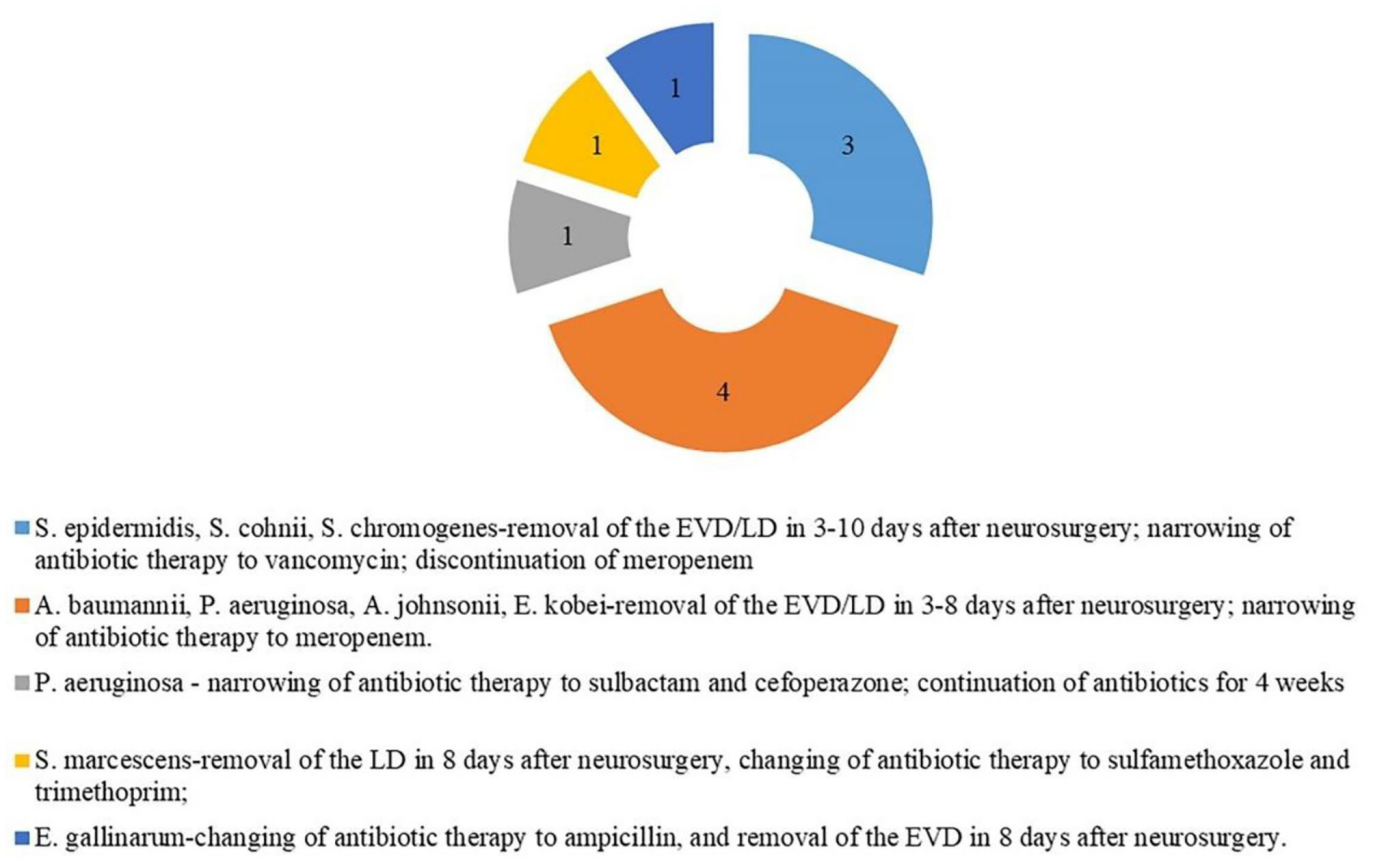
Clinical effect of the results of mNGS (10 of 22 cases diagnosed by mNGS only).

In the cVM group, the positive mNGS detection rate was 61.54% (24/39). Inconsistent positive agents revealed by conventional tests were seen in 7 (17.95%) patients and mainly included *Moraxella osloensis, Escherichia coli, Klebsiella Pneumoniae, Staphylococcus haemolyticus, Staphylococcus xylosus*, and *Staphylococcus hominis*. Moreover, mNGS effectively identified coinfections from multiple bacteria. Among 49 patients with sVM, mNGS identified bacterial pathogens in 22 patients (44.90%) that were negative based on the conventional method. Despite such discrepancy, 10 of those 22 patients showed probable clinical responses (Figure 3). All 27 CSF samples in the nVM group were concordant between the conventional method and mNGS (Table 2).

**Table 2:** The results of mNGS and conventional microbiological testing.

**cVM (39)**	**mNGS**	**Conventional microbiological testing**	**sVM (49)**	**mNGS**
**/number**			**/number**	
1	*A. baumannii*	*A. baumannii*	1	*A. sclerotioniger*
2	*S. epidermidis*	*S. epidermidis*	2	*K. palustris*
3	*S. epidermidis*	*S. epidermidis*	3	*S. marcescens*
4	*S. epidermidis*	*S. epidermidis*	4	*R. mucosa*
5	*S. epidermidis*	*S. epidermidis*	5	*A. johnsonii*
6	*S. epidermidis*	*S. epidermidis*	6	*A. baumannii*
7	*S. epidermidis*	*S. epidermidis*	7	*P. aeruginosa*
8	*S. epidermidis*	*S. epidermidis*	8	*P. aeruginosa*
9	*S. epidermidis*	*S. epidermidis*	9	*S. maltophilia*
10	*E. aerogenes*	*E. aerogenes*	10	*E. kobei*
11	*K. pneumoniae*	*K. pneumoniae*	11	*S. equi*
12	*S. aureus*	*S. aureus*	12	*S. oralis*
13	*S. aureus*	*S. aureus*	13	*C. testosteroni*
14	*S. aureus*	*S. aureus*	14	*B. multivorans*
15	*E. durans*	*E. durans*	15	*E. gallinarum*
16	*S. hominis*	*S. hominis*	16	*C. indologenes*
17	*C. albicans*	*C. albicans*	17	*S. chromogenes*
18	*C. albicans*	*C. albicans*	18	*S. epidermidis*
19	*S. maltophilia*	*S. maltophilia*	19	*S. cohnii*
20	*A. baumannii*	*A. baumannii*	20	*M. restricta*
21	*A. baumannii*	*A. baumannii*	21	*M. restricta*
22	*A. baumannii*	*A. baumannii*	22	*C. tuberculosis*
23	*S. hominis*	*S. hominis*		
24	*A. baumannii*	*A. baumannii*		
25	*P. bivia*	*M. osloensis*		
26	*P. buccalis*	*E. coli*		
27	*L. rhamnosus*	*K. pneumoniae*		
28	/	*E. faecalis*		
29	/	*E. faecalis*		
30	*C. testosteroni*	*S. haemolyticus*		
31	*C. amalonaticus*	*S. haemolyticus*		
32	/	*V. Streptococci*		
33	/	*S. shominis*		
34	/	*S. capitatum*		
35	/	*S. capitatum*		
36	/	*S. warneri*		
37	*H. parahaemolyticus*	*S. haemolyticus*		
38	*S. aureus*	*S. hominis*		
39	*P. bivia*	*S. hominis*		

### Unique Reads for Pathogens Detected by mNGS

In the cVM group, the median number of unique reads detected by mNGS was 248.5 (range, 39.5–8,043.5), while the median was 4 (range, 3–6) in patients with sVM, thus demonstrating a marked difference between these groups (*P* < 0.01; Table 3).

**Table 3:** Unique reads for pathogens detected by mNGS.

**Microorganism**	**Unique reads**	**Microorganism**	**Unique reads**	***P*-value**
cVM-*A. baumannii*-1	250,078	sVM-*A. sclerotioniger*-1	10	*P* < 0.01
cVM-*S. epidermidis*-2	78	sVM-*K. palustris*-2	7	
cVM-*S. epidermidis*-3	149	sVM-*S. marcescens*-3	8	
cVM-*S. epidermidis*-4	13	sVM-*R. mucosa*-4	13	
cVM-*S. epidermidis*-5	3,496	sVM-*A. johnsonii*-5	3	
cVM-*S. epidermidis*-6	348	sVM-*A. baumannii*-6	3	
cVM-*S. epidermidis*-7	5,451	sVM-*P. aeruginosa*-7	4	
cVM-*S. epidermidis*-8	15,821	sVM-*P. aeruginosa*-8	3	
cVM-*S. epidermidis*-9	12	sVM-*S. maltophilia*-9	7	
cVM-*E. aerogenes*-10	955	sVM-*E. kobei*-10	4	
cVM-*K. Pneumoniae*-11	512,31	sVM-*S. equi*-11	3	
cVM-*S. aureus*-12	17	sVM-*S. oralis-*12	6	
cVM-*S. aureus*-13	434	sVM-*C. testosteroni*-13	3	
cVM-*S. aureus*-14	70	sVM-*B. multivorans*-14	5	
cVM-*E. durans*-15	35,244	sVM-*E. gallinarum*-15	3	
cVM-*S. hominis*-16	4	sVM-*C. indologenes*-16	3	
cVM-*C. albicans-*17	49	sVM-*S. chromogenes*-17	3	
cVM-*C. albicans-*18	41	sVM-*S. epidermidis*-18	5	
cVM-*S. maltophilia-*19	35,862	sVM-*S. cohnii*-19	6	
cVM-*A. baumannii*-20	120,122	sVM-M. restricta*-*20	3	
cVM-*A. baumannii*-21	3,785	sVM-*M. restricta*-21	4	
cVM-*A. baumannii-*22	43	sVM-*C. tuberculosis*-22	5	
cVM-*S. hominis*-16	35			
cVM-*A. baumannii*-24	24			
cVM-Median (IQR)	248.5 (39.5, 8,043.5)	sVM-Median (IQR)	4 (3, 6)	

### Antibiotic Resistance Gene Detection

Using mNGS, antibiotic resistance was identified in three CSF samples from cVM patients. Such resistance was imparted by *A. baumannii* and one instance of *S. maltophilia*. The results revealed the presence of several antibiotic resistance genes, including *adeIJK, adeFGH*, and *AbaQ*, which cause resistance to fluoroquinolones or tetracyclines. Additionally, *smeABC* and *smeDEF* genes that cause resistance to multiple antibiotics were also identified.

## Discussion

To our knowledge, the present study is the first to evaluate the use of mNGS for pathogen detection in a large prospective cohort of patients with EVD/LD-associated VM. Patients were divided into three subgroups based on routine clinical test results. The effectiveness and efficiency of mNGS were compared to a conventional microbiological identification method using CSF samples. In addition, we proposed new criteria for the determination of positive specimens according our detection methods. Our study showed that mNGS can provide much quicker and more accurate etiologic pathogen identification results than the conventional microbiological identification method.

VM is an acute complication associated with neurosurgery and may lead to permanent adverse outcomes if not managed properly (Beer et al., 2008). The incidence of VM rates was reported to range from 2 to 45% (Dorresteijn et al., 2019), and the predisposing factors for VM include non-adherence to rigid insertion and maintenance protocols, leakage of CSF, catheter irrigation, and the frequency of EVD or LD manipulation. Meanwhile, our study indicated the same results in cVM and sVM (39 and 49 of 102 patients, respectively). The prevention and management of EVD/LD-associated VM are great clinical challenges, particularly due to the difficulty in detecting pathogens in a timely manner and the re-emergence of infections caused by multidrug-resistant pathogens and emergent invasive neurosurgical procedures (Mayhall et al., 1984). Approximately 50% of meningoencephalitis cases remain undiagnosed, despite extensive clinical laboratory testing (Glaser et al., 2006). Thus, novel technologies, such as mNGS, are particularly important for the diagnosis and evaluation of VM.

In our study, untargeted shotgun mNGS analyses were performed to sequence the entirety of the DNA and/or RNA, rather than using specific primers or probes (Quince et al., 2017). This method was successfully performed on 102 samples. Although insufficient starting DNA concentrations in CSF samples might limit microbial detection due to insufficient sequencing data or sequencing failure, our optimized mNGS pipeline identified high numbers of pathogen reads from such samples. mNGS was also able to distinguish a broader spectrum of pathogens compared to the conventional culture method. As shown by several studies, mNGS has also successfully diagnosed rare (Wilson et al., 2014), novel (Hoffmann et al., 2015), and atypical infectious etiologies (Mongkolrattanothai et al., 2017; Wilson et al., 2018) of encephalitis. In this study, among the 22 samples of sVM diagnosed by mNGS, the causative pathogen was either not considered by clinicians or had been tested negative by conventional testing. Remarkably, 10 of those 22 patients showed mNGS-guided clinical responses. For example, a patient infected with *P. aeruginosa* (according to mNGS) experienced fever 10 days after neurosurgery. Empirical antimicrobial therapy administered by clinicians did not relieve infection-related symptoms, such as headache and a low level of consciousness. However, the mNGS result guided the empirical antimicrobial therapy of this patient, indicating its clinical utility.

Moreover, there were 24 cases co-diagnosed by mNGS and the conventional method. The results of mNGS were valuable in reassuring the results from the conventional method, and in the detection or exclusion of coinfections, particularly in patients receiving high-grade prophylactic antibiotic administration. Those findings highlighted the outstanding advantage of mNGS, in that it does not rely on *a priori* selection of targeted pathogens, but rather, it detects several potential infectious agents in a single assay (Goldberg et al., 2015). Thus, mNGS was useful for diagnostic testing of CSF samples, regardless of sample volume and availability. Collectively, a combination of mNGS and conventional testing of CSF samples improved the detection of infections (Xing et al., 2020).

Among patients with cVM, mNGS reported negative results in eight cases. However, a negative mNGS sample did not necessarily mean the patient was negative for a clinical infection (Schlaberg et al., 2017), as the prior use of antibiotics could affect pathogen detection. In this study, the majority of patients with cVM received prophylactic antibiotics and/or empirical antibiotics before the CSF samples were obtained for mNGS testing. Additionally, for samples that had undergone multiple freeze–thaw cycles, the diagnostic yield could be decreased. Finally, we observed that low numbers of unique reads were usually observed in the false-negative results. In the cVM patient cohort, mNGS identified different pathogens in seven patients, potentially due to factors including the contamination of pathogen DNA across samples during mNGS library preparation, low-complexity sequences matching low-quality reads from the sample, misannotated species, or contaminants from database entries, sequencing adaptors, or vectors, colonization (Zhang et al., 2019). Elevated levels of human circulating free DNA (cfDNA) backgrounds in samples had only little effects on the sensitivity and precision, while the level of environmental contamination influenced sensitivity (Blauwkamp et al., 2019). It is worth noting that when using mNGS, repeated sequencing may be necessary when inconsistent results are observed.

Removing false-positive results was a primary challenge for mNGS analysis. False-positive results are likely to mislead therapeutic decision making, and clinicians are cautious when interpreting the sequences of bacteria that are commonly found in hospital environments. Thus, great effort was made to reduce the influence of false-positive results. The complexity of mNGS analysis required us to reduce the potential of false-positive results including in the sampling process, laboratory practices, reagents, environment, and skin or other commensal flora (Laurence et al., 2014; Lusk, 2014; Strong et al., 2014; Hocquet et al., 2016). For example, we took extreme care with sample handling to avoid cross-contamination (Chiu and Miller, 2019). We developed a strict laboratory workflow, whereby we required highly trained personnel to perform analyses and focused on minute amounts of exogenous nucleic acids introduced during specimen collection, aliquoting, nucleic acid extraction, and library preparation. We also ensured that laboratory surfaces, consumables, and reagents were DNA free. We also formulated strict bioinformatic criteria for calling positive results.

The number of unique mNGS reads in the sVM group was significantly lower than that of the cVM group, potentially due to the prior use of antibiotics or the development of diseases that affected pathogen concentration. As it is difficult to identify bacteria or fungi in extremely low concentrations by conventional microbiological measures, mNGS is useful due to its high sensitivity.

The most common causative bacterial pathogens of VM are skin commensals, such as coagulase-negative staphylococci, *S. aureus, E. coli, A. baumannii*, and *K. pneumoniae* (Dorresteijn et al., 2020). This was similar to the findings of our study, where *S. epidermidis, A. baumannii*, and *S. aureus* were the primary pathogens causing VM. The information provided by mNGS was leveraged to identify antibiotic resistance mechanisms from CSF samples to evaluate disease risk (Stefan et al., 2016). The genes, *adeIJK, adeFGH, AbaQ, adeABC*, and *adeL* were identified in three CSF samples where cVM was caused by *A. baumannii*. Those antibiotic resistance genes cause resistance to fluoroquinolones and tetracyclines (Damier-Piolle et al., 2008; Pérez-Varela et al., 2018). The *S. maltophilia* antibiotic resistance genes, *smeABC* and *smeDEF*, were also found in one CSF sample (Li et al., 2002). This information facilitated the implementation of rational antibiotic administration guidelines. The detrimental effects of delays in the administration of antibiotics are widely known from the management of meningitis (van de Beek et al., 2012). Thus, if rapid bacterial identification is performed by mNGS, a timelier adjustment in antibiotics can be achieved and might lead to a better prognosis for VM patients.

Even though mNGS detected several pathogens that complemented conventional testing and played an important role in the guidance of clinical EVD/LD-associated VM treatment, there are some limitations to this technology. The financial cost of mNGS is higher than that of conventional testing. A number of factors also need to be ensured for accurate detection, including the quality of the available CSF samples, including the timing of their collection relative to symptom onset, whether the sample was adequately handled to ensure sterility, and whether the available DNA libraries offer wide coverage.

There are also several limitations to this research. First, this study was a single-center study with a single cohort of patients, thus resulting in a small sample size, especially after stratification of patients according to the VM criteria. Second, antibiotic resistance genes were only detected in four samples, which cannot indicate the ability of mNGS in resistance gene detection. Finally, we did not conduct real-time quantitative PCR to verify the mNGS results due to the insufficient volumes of CSF. Thus, clinical verification (clinical diagnosis, antibiotic treatment, etc.) was used as a substitute. With continuous development and optimization of mNGS technology, and stricter standards for sample collection and storage, we will perform a multicenter prospective study to better evaluate the use of mNGS in VM.

## Conclusions

Our findings revealed that mNGS of CSF is a practical tool for the diagnostic evaluation of patients with VM. Compared with traditional diagnostic tools, mNGS is a rapid and accurate method for pathogen identification and disease diagnosis. This study highlights the feasibility of testing CSF samples with mNGS as a diagnostic tool for EVD/LD-associated VM to guide timely and targeted treatments.

## Data Availability Statement

The data analyzed in this study is subject to the following licenses/restrictions: no restrictions. Requests to access these datasets should be directed to Guojun Zhang. zgjlunwen@163.com.

## Ethics Statement

The studies involving human participants were reviewed and approved by this study was approved by the ethical committee of Beijing Tiantan Hospital and Capital Medical University (Approved Number: KY-2019-095-03). The patients/participants provided their written informed consent to participate in this study.

## Author Contributions

GZha and GZhe conceived and designed the study. LQ retrieved the literature and drafted the manuscript. YS and FL reviewed the literature and edited the manuscript. YW, MM, YZ, and YWS participated in the study design and editing of the manuscript. All authors read and approved the final manuscript.

## Conflict of Interest

YWS was employed by Nanjing Geneseeq Technology Inc. The remaining authors declare that the research was conducted in the absence of any commercial or financial relationships that could be construed as a potential conflict of interest.
